# Portuguese Version of the Intentional Non-Adherence Scale: Validation in a Population of Chronic Pain Patients

**DOI:** 10.3389/fphar.2021.681378

**Published:** 2021-07-19

**Authors:** Rute Sampaio, Mariana Cruz, Simão Pinho, Cláudia Camila Dias, John Weinman, José M. Castro Lopes

**Affiliations:** ^1^ Departamento de Biomedicina, Faculdade de Medicina da Universidade do Porto, Porto, Portugal; ^2^ Centro de Investigação em Tecnologias e em Serviços de Saúde (CINTESIS), Porto, Portugal; ^3^ Departamento de Medicina da Comunidade, Informação e Decisão em Saúde, Faculdade de Medicina da Universidade do Porto (MEDCIDS), Porto, Portugal; ^4^ Kings College London, Institute of Pharmaceutical Sciences, London, United Kingdom; ^5^ Instituto de Investigação e Inovação em Saúde (I3S), Universidade do Porto, Porto, Portugal

**Keywords:** medication adherence, chronic pain, reliability, validity, factor analysis

## Abstract

**Objective:** Despite the effectiveness of pain medicines, nonadherence to prescribed medication remains a major problem faced by healthcare systems. The aim of present study was to perform the translation, cultural adaptation, and validation of the Intentional Non-Adherence Scale (INAS) for the European Portuguese language in a sample of chronic pain patients.

**Methods:** A Portuguese version of the INAS scale was constructed through a process of translation, back translation, and expert’s panel evaluation. A total of 133 chronic pain patients were selected from two chronic pain clinics from tertiary hospitals in Porto, Portugal. The protocol interview included the assessment of pain beliefs (PBPI), beliefs about medicine (BMQ), medication adherence (MARS-P9), and two direct questions about adherence previously validated.

**Results:** The internal consistency in all subscales was *α* = 0.902 for testing treatment; *α* = 0.930 for mistrust treatment; *α* = 0.917 for resisting treatment; and *α* = 0.889 for resisting illness. Exploratory and confirmatory factor analysis revealed a four-factor structure that explained 74% of the variance. The construct validity of the INAS was shown to be adequate, with the majority of the previously defined hypotheses regarding intercorrelations with other measures confirmed.

**Conclusion:** The Portuguese version of INAS could be a valuable and available instrument for Portuguese researchers and clinicians to assess the intentional nonadherence determinants during the management of chronic pain.

## Introduction

Chronic pain (CP) is the main cause of suffering and disability in the world (Salduker et al., 2019; Treede et al., 2019; Baronian and Leggett, 2020), affecting 19% of Europeans (Breivik et al., 2006). In Portugal, this condition is particularly prevalent as it affects 37% of the population (Azevedo et al., 2012). Pain is one of the main reasons for the use and overuse of health services, resulting in an annual cost of approximately €440 billion in Europe (Federation, 2017).

An extremely important factor for the success of chronic pain treatment is the patient’s adherence to pharmacological treatment (Vrijens et al., 2012; Azevedo et al., 2017). However, recent data reveal that nonadherence in chronic pain patients is very prevalent, with a rate of about 40%, and that it is associated with increased morbidity, mortality, and avoidable costs (Iuga and McGuire, 2014; Timmerman et al., 2016; Cutler et al., 2018). Patients’ beliefs and concerns greatly impact their adherence behavior and addressing them is an essential part of tackling this issue (Rosser et al., 2011; Timmerman et al., 2016; Sampaio et al., 2020).

Given the great complexity and heterogeneity of human behavior, it is extremely difficult to reliably predict patients’ medication adherence (Mukhtar et al., 2014; Sampaio et al., 2020). Moreover, notwithstanding being historically viewed as a homogenous entity, unintentional nonadherence occurs when the patient fails to follow a recommended therapeutic intervention, without making a conscious decision (Wroe, 2002; Lehane and McCarthy, 2007; Clifford et al., 2008). Intentional nonadherence is, conversely, an active behavior, a deliberate decision to discontinue, skip, or alter a prescribed therapy (Lehane and McCarthy, 2007; Clifford et al., 2008; Mukhtar et al., 2014).

In recent years, several adherence self-reporting tools have emerged; despite this, there is still no consensus on what the gold standard is and, to adequately evaluate adherence, one must apply diverse strategies (Nguyen et al., 2014; Stirratt et al., 2015; Monnette et al., 2018). However, studies have shown that some of the most widely used adherence scales tend to greatly overestimate adherence as they are very vulnerable to the social desirability bias (Stirratt et al., 2015; Brown et al., 2016; Monnette et al., 2018). As such, it follows the rigorous development and testing of tools that promote a blame-free environment and, particularly, differentiate between intentional and unintentional nonadherence, which is of paramount importance (Stirratt et al., 2015).

The Intentional Non-Adherence Scale (INAS) is a tool developed by Weinman et al. (2018) to assess intentional nonadherence to any prescribed medications (Weinman et al., 2018). The questionnaire expressly establishes a judgment-free stance in its introduction and consists of 22 items describing reasons for nonadherence (Weinman et al., 2018). Regardless of its potential, to our knowledge, INAS has yet to be translated in other languages.

The aim of this study was to provide a translated and culturally adapted Portuguese version of the INAS for evaluating treatment adherence as well as assessing its applicability, reliability, and validity in a population of chronic pain patients. Nonadherence ubiquity across all therapeutic fields and the importance of treatment adherence in chronic pain make it appropriate to validate the questionnaire in the chosen population.

## Materials and Methods

### Translation and Cultural Adaptation

The guidelines for translation and adaptation of self-report instruments defined by the ISPOR Task Force for Translation and Cultural Adaptation (Wild et al., 2005) were followed for the development of the Portuguese version of INAS. The English version of INAS was first translated into Portuguese language by two independent Portuguese native speakers. The back-translation was performed by a professional service, unaware of the purpose of the research. A panel of six experts (a health psychologist, a professor of pain medicine, a methodologist with experience in psychometrics, a nurse, and two anesthesiologists working in the field of chronic pain) assessed the similarity between versions and examined and resolved all inconsistencies and ambiguities by consensus. Finally, the back translation was approved by Professor John Weinman. To appraise the comprehension of the language and wording, the revised preliminary Portuguese version of INAS was applied to a pilot sample of 10 chronic pain patients prescribed with pharmacotherapy and native in Portuguese. The final Portuguese version of the INAS was defined and used in the validation process.

### Participants

A total of 133 CP outpatients were selected from two chronic pain clinics from tertiary hospitals in Porto, Portugal. The inclusion criteria were: at least 18 years old, confirmed CP for more than 3 months, able to communicate in Portuguese language, the absence of psychiatric and cognitive disorders precluding the interviews, and willingness to participate in the study. The patient selection was performed using a consecutive sampling scheme, and the sample size calculation was performed aiming to estimate reliability and validity coefficients with a maximum margin of error of ±0.1 and a confidence level of 95% (Streiner and Norman, 1995).

The study protocol was approved by the hospital review boards and ethics committee of the two hospitals involved.

### Instruments

#### Intentional Non-Adherence Scale

The INAS aims to assess different components of intentional nonadherence and to identify some of the reasons why patients may intentionally stop taking their medication as prescribed (Weinman et al., 2018). The authors attempted to identify intentional factors that get in the way of adhering to medical treatment, from the patient’s point of view (Weinman et al., 2018). The questionnaire presents a judgment-free stance, expressed in its introduction. After the brief introduction, patients are presented with 22 reasons why people cease to take their medications as prescribed and are asked to rate each reason, regarding the last 6 months, on a five-point Likert scale: *1 = strongly disagree, 2 = disagree, 3 = neutral, 4 = agree, 5 = strongly agree* (Weinman et al., 2018). A factor analysis of the English language version revealed two subscales, which were labeled as Resisting Illness *(RI)—the decision not to take treatment with not wanting to be reminded of one’s illness, the association of medication with illness, and the desire to feel “normal”*; *and* Testing Treatment (TT)—*the reasons for not taking treatment based on the person’s attempts to see if they can get away with taking less or no treatment* (Weinman et al., 2018). Both subscales had high internal reliability (0.95 and 0.93, respectively). This original tool can be used to assess reasons for nonadherence to any prescribed drug, regardless of the health condition, while also being a patient-friendly and a reliable scale that will make it possible to design novel interventions directed toward intentional nonadherence (Weinman et al., 2018).

#### Portuguese Pain Beliefs and Perception Inventory

The Pain Beliefs and Perception Inventory (PBPI-P) assesses the degree of agreement with a statement referring to pain-related beliefs or perceptions (Williams et al., 1994). The PBPI-P is a self-administered questionnaire, composed of 16 items with different statements about common pain-related beliefs and perceptions. In each item a bipolar Likert scale with four levels (from −2 to +2 and with no zero; anchored to the following descriptors – “strongly disagree,” “disagree,” “agree,” and “strongly agree”) is used. The Portuguese version (PBPI-P) revealed a four-factor structure (Azevedo et al., 2016). The first two factors refer to beliefs and perceptions associated with the impact of time and persistence of pain; and these were concerned with beliefs that pain is and will be enduring (*Permanence and Constancy*). The third factor is associated with the mysterious or enigmatic nature of pain and its causes, and includes statements about the poorly understood nature of pain (*Mystery*). The fourth factor is associated with beliefs and perceptions of self-blame in relation with pain (*Self-blame*). The total score is obtained by dividing the total sum by the number of items. Higher scores indicate greater endorsement of the beliefs and perceptions.

#### Beliefs About Medicine Questionnaire

The BMQ has proven to be useful to assess patients’ beliefs associated with nonadherence to treatments and providing information about patients’ actual medication taking behavior (Horne et al., 1999). The Portuguese version of BMQ is an 11-item scale scored on a five-point Likert scale (1 = strongly disagree, 2 = disagree, 3 = uncertain, 4 = agree, and 5 = strongly agree) (Salgado et al., 2013). It comprises two subscales: a five-item Necessity scale, to assess beliefs about the necessity for prescribed medication (Specific-Necessity), and a six-item Concerns scale, to assess beliefs about the danger of dependence and long-term toxicity and the disruptive effects of medication (Specific-Concerns). A higher score represents the greater patient’s belief in necessity vs. concerns concept. A necessity-concerns differential can also be calculated by subtracting the Concerns subscale scores from the Necessity subscale scores, such that the higher differential scores indicate higher perceived necessity and/or lower concerns, thereby representing lower likelihood of intentional nonadherence. The Cronbach’s alpha coefficient for the Portuguese BMQ-Specific is 0.70, and 0.76 and 0.67 for the Necessity and Concerns subscales, respectively. The BMQ also includes two scales assessing general beliefs about medicines (Harm; Overuse). These subscales are composed by four-item each and also scored on a five-point Likert scale (1 = strongly disagree, 2 = disagree, 3 = uncertain, 4 = agree, and 5 = strongly agree). Patients who believed that medicines in general are intrinsically harmful would be more likely to believe that it is better to avoid taking them and to consider themselves to be susceptible to potential adverse effects of medication. The overuse subscale is the notion that medicines are over-prescribed by doctors who place too much trust in them.

#### Medication Adherence Rating Scale

The MARS evaluates nonadherence in a nonthreatening way, where questions are posed as a negative statement to minimize social desirability bias (Mora et al., 2011). MARS is available in several versions (with 4, 5, 9, and 10 items), languages (English, German, and Arabic), and in a range of long-term conditions (asthma, cardiovascular diseases, chronic obstructive pulmonary disease, diabetes, inflammatory bowel disease, depression, and bipolar disease (Horne and Weinman, 2002; George et al., 2005; Fialko et al., 2008; Clatworthy et al., 2009; Horne et al., 2009; Tibaldi et al., 2009; Mahler et al., 2010; Mora et al., 2011; Salt et al., 2012; Alsous et al., 2017). We used a recently published validated Portuguese version, called MARS-P9, which reveals excellent psychometric properties (Sampaio et al., 2019). Internal consistency measured by Cronbach’s alpha coefficient was (*α* = 0.84) superior than that to a sample of asthma patients (*α* = 0.83) (Ohm and Aaronson, 2006) and with a sample of rheumatoid arthritis patients (*α* = 0.77) (Salt et al., 2012). Concerning the factorial validity of MARS-9P, the two-factor structure found in the present study falls into two categories, assuming an intentional and an unintentional nonadherence behavior (Mora et al., 2011). Unintentional nonadherence was only assessed with one item as observed in other studies (Mora et al., 2011; Salt et al., 2012). Responses are recorded on a five-point Likert scale, ranging from 1 (always) to 5 (never), and only one item (Timmerman et al., 2016) is inverted. Higher scores indicate higher adherence.

### Data Collection Methods

From March 2019 to January 2020, 133 CP patients were enrolled after their consultation with the attending physician. Patients followed a standardized protocol that included a first face-to-face interview performed by one of the two trained interviewers, and with the attending physician and nurse collaboration to access the clinical file of the patient. The same protocol was repeated after one month by telephone interviewing performed by the same interviewer that was with patient at the first assessment moment. The protocol interview included sociodemographic questions, the Portuguese versions of the questionnaires previously described (PBPI, BMQ, MARS-P9, and INAS), and two direct questions about adherence previously validated (Sampaio et al., 2020) (“Is there any medicine that you have decided not to take?” and “Is there any medicine that you have decided not to take as prescribed?”). All participants were initially informed about the study objectives and the selection and data collection procedures, and after properly answering their questions, they were asked to sign the informed consent form, before they were included into the study.

#### Statistical Analysis and Assessment of Reliability and Validity

A descriptive analysis of the general characteristics of the sample was performed. Continuous variables were summarized using the mean, standard deviation (SD), and 95% confidence intervals (CI). Summary statistics were presented for each item and subscale, including the proportion of scores in the extremes of the scales, to assess the ceiling and floor effects (Nunnally, 1994). The assessment of the psychometric properties of the INAS included test–retest reliability, internal consistency, factorial validity, and construct (convergent and discriminant) validity following the internationally recommended standards (Nunnally, 1994; McDowell, 2006; Fayers and Machin, 2007).

Analysis of internal consistency was performed by assessing the Cronbach’s alpha statistic, the Cronbach’s alpha coefficient when the items were deleted, and the item-total correlation. The test-retest reliability was assessed by the estimation of agreement between the baseline and the 1 month assessments, using appropriate statistics (intraclass correlation coefficient). Assessments of the construct (convergent and discriminant) validity was performed by calculating and evaluating the correlations, defined by a set of previously developed theoretical hypotheses, between the subscales of INAS and MARS-9P, BMQ, and PBPI [measures of the same constructs used in the original study (22)]. The existence of significant correlations was assumed, namely, the beliefs and perceptions about pain, and its treatment could be associated to the intentional constructs of nonadherence to the pain treatment.

As general practical rules, interpretation of the reliability and correlation coefficients was based, respectively, on the quantitative criteria and qualitative descriptors defined by Landis and Koch (1977) and Cohen (1988); interpretation of the Cronbach’s alpha coefficient measures followed the recommendations by Nunnally (1994) and Bernstein.

Models of exploratory factor analysis were defined using principal components analysis for factor extraction (Thompson, 2004; Brown, 2006). Orthogonal varimax rotations were applied to improve the interpretation of factors. The number of factors was selected taking into account the Kaiser’s criterion (eigenvalues larger than one) and the total of variance explained (at least above 50%).

Confirmatory factor analysis was performed using structural equation models (SEMs), with parameter estimation based on maximum likelihood methods using jamovi software program (version 1.1.3). A multiple index was computed to assess the model fit: 1) the comparative fit index (CFI) with a good fit for values above 0.9; 2) the Tucker–Lewis index (TLI) with a good fit for values above 0.95; and 3) root-mean-square error of approximation (RMSEA) and its respective 90% confidence interval with a good fit for values between 0.05 and 0.08.

For all hypothesis tests, a significance level of 0.05 was defined. Statistical analysis was performed using the software Statistical Package for the Social Sciences (SPSS) v. 24.0; and jamovi software program (version 1.1.3) for SEMs.

## Results

### Translation and Cultural Adaptation

A panel of six experts performed the assessment and reconciliation of translations and cultural adaptation of the INAS, considering the minutiae of Portuguese language and the particular characteristics of CP patients. Consensus among all panel members was reached for all items. A preliminary version of INAS was tested on a sample of 10 CP patients to confirm the applicability of the questionnaire and the quality of the translation and cultural adaptation. The back translation was approved by the author of INAS, Professor John Weinman. Only one question was raised concerning the translation of item 19, namely, a misinterpretation of the back-translation, and it was agreed to add “…to take it” to finish the item: “Because it is good not have to remember to take it.”

### General Characteristics of the Sample

The participants (*n* = 133) were 95 females and 38 males, between 27 and 84 years old, with a mean age of 57 years (SD = 12.7). The majority had completed four or fewer years of education (33%), 29% attended basic second and third cycles, 20% attended high school, and 18% have a higher education degree. Almost 34% of patients were retired, 34% had a full-time job, and 13% were on sick leave. According to the Chronic Pain Syndromes Classification of the International Association for the Study of Pain—IASP (International Association for the study of Pain, 2019), the distribution of the main pain diagnoses was as follows: oncologic (43.6%), musculoskeletal (33.9%), neuropathic (36.1%), and post-surgical or post-traumatic (17.3%). The majority of patients had a single pain diagnosis (68%), 30% had two pain diagnoses, and only 1.5% had three pain diagnoses.

Summary statistics and missing data for items and subscales of INAS are described in Table 1. There are floor effects in every item, but no ceiling effects. There were no missing data for almost all items, except for item 11 with a value of 0.8% (not shown on the table).

**TABLE 1:** Descriptive analysis and internal consistency of items and subscales of Portuguese version of INAS (*n* = 133).

Items	Mean	SD	Floor effect*^a^* (%)	Ceiling effet*^b^*	∝	Item-total correlation	∝If item deleted	Test-retest reliability ICC (95%CI)
Testing treatment subscale	5.7	3.6	53	1%	0.902	—	—	0.545 (0.349–0.682)
To see if my illness/problem is still there	1.7	1.2	68	2%	—	0.667	0.967	—
To see if I can do without it	2.1	1.4	56	5%	—	0.882	0.790	—
To see if I really need it	2.0	1.4	59	5%	—	0.893	0.780	—
Mistrust treatment	3.4	2.3	63	3%	0.930	—	—	0.550 (0.356–0.685)
Because I am not convinced that the medicine is really right for me	1.8	1.2	65	5%	—	0.870	—	—
Because I am not sure that the doctor chose the right medicine for me	1.7	1.2	67	3%	—	0.870	—	—
Resisting treatment	12.7	7.4	46	2%	0.917	—	—	0.607 (0438–0.725)
To give my body a rest from the medicine	1.8	1.3	65	5%	—	0.688	0.911	—
Because the medicine is harsh on my body	1.9	1.3	59	5%	—	0.739	0.905	—
Because I don’t like the medicine to accumulate in my body	1.7	1.2	65	4%	—	0.791	0.900	—
Because my body is sensitive to the effects of medicine	2.0	1.4	59	8%	—	0.737	0.906	—
Because I don’t like the side effects	2.1	1.5	58	11%	—	0.776	0.902	—
Because I don’t like chemicals in my body	1.6	1.2	71	5%	—	0.816	0.898	—
Because it may affect the body’s own natural healing processes	1.6	1.1	71	4%	—	0.706	0.909	—
Resisting illness	9.2	5.0	56	2%	0.889	—	—	0.568 (0.383–0.698)
Because I think I am on too high a dose	1.5	1.1	74	5	—	0.573	0.891	—
Because I worry about becoming dependent on my medicine	1.7	1.2	69	6%	—	0.698	0.873	—
Because I want to think of myself as a healthy person again	1.6	1.1	66	2%	—	0.826	0.850	—
Because it reminds me that I have an illness	1.5	1.0	74	2%	—	0.763	0.862	—
Because I want to lead a normal life again	1.5	1.0	72	3%	—	0.775	0.859	—
Because it is good not to have to remember	1.4	0.9	76	2%	—	0.634	0.881	—

a Percentage of subjects scoring in the minimum of the scale (floor effect).

b Percentage of subjects scoring in the maximum of the scale (ceiling effect).

SD, standard deviation; ∝, Cronbach’s alpha coefficient; ICC, intraclass correlation coefficient; and CI, confidence interval.

### Internal Consistency and Test-Retest Reliability

The analysis of internal and test-retest reliability of INAS is presented in Table 1. Internal consistency was excellent in all subscales: *α* = 0.902 for testing treatment, *α* = 0.930 for mistrust treatment, *α* = 0.917 for resisting treatment, and *α* = 0.889 for resisting illness. Test-retest reliability coefficients reveal poor reliability (bellow 0.60) for all subscales.

### Factor Analysis

Construct validity was assessed by performing factor analysis with principal components and varimax rotation methods (Kaiser, 1958). The suitability of the factor analysis by checking the existence of significant correlations between the items was confirmed by Kaiser‐Meyer‐Olkin (KMO = 0.858) and by the Bartlett’s sphericity test (QQ = 2074.657; gl = 153; and *p* < 0.001). Four factors with eigenvalues greater than 1.0 were extracted accounting for 74% of total variance. It yielded eighteen items with component loadings greater than 0.50. Factor loadings are shown in Table 2. The first factor explained 51.21% of total variance and covered the five items of the original INAS. Additionally, it included item-5 that was removed from the original scale. A second factor emerged and explained 10.22% of total variance, labeled as “Resisting treatment”. It included seven items showing some reluctance in taking the treatment. The third factor explained 6.7% of the total variance and covered the three items of the original INAS. Finally, a fourth factor emerged and explained 6.2% of total variance and the included two items labeled “Mistrust treatment” as they reveal some suspicion if treatment is accurate.

**TABLE 2:** Exploratory factor Analysis structure of the INAS: loading for each factor and each item in the model with four factors after an orthogonal varimax rotation and factor extraction using principal components (*n* = 133).

Items	Factors*^a^*			
	1	2	3	4
*Testing treatment*	—	—	—	—
To see if my illness/problem is still there	0.306	0.056	0.788	0.086
To see if I can do without it	0.201	0.258	0.865	0.093
To see if I really need it	0.224	0.219	0.879	0.028
*Mistrust treatment*	—	—	—	—
Because I am not convinced that the medicine is really right for me	0.175	0.244	0.093	0.908
Because I am not sure that the doctor chose the right medicine for me	0.135	0.235	0.062	0.899
*Resisting treatment*	—	—	—	—
To give my body a rest from the medicine	0.277	0.537	0.531	0.118
Because the medicine is harsh on my body	0.444	0.570	0.188	0.326
Because I don’t like the medicine to accumulate in my body	0.455	0.607	0.319	0.207
Because my body is sensitive to the effects of medicine	0.206	0.826	0.061	0.217
Because I don’t like the side effects	0.226	0.858	0.104	0.133
Because I don’t like chemicals in my body	0.340	0.713	0.335	0.171
Because it may affect the body’s own natural healing processes	0.246	0.604	0.338	0.273
*Resisting illness*	—	—	—	—
Because I think I am on too high a dose	0.586	0.221	0.094	0.373
Because I worry about becoming dependent on my medicine	0.648	0.232	0.276	0.285
Because I want to think of myself as a healthy person again	0.834	0.244	0.186	0.162
Because it reminds me that I have an illness	0.812	0.171	0.277	0.083
Because I want to lead a normal life again	0.685	0.382	0.378	0.070
Because it is good not to have to remember	0.657	0.320	0.190	−0.033
*Percentage of total variance explained for each factor*	51.21	10.22	6.70	6.20

a Percentage of total variance explained for the four extracted factors = 74%; KMO = 0.858, and Bartlett’s Test of Sphericity <0.001. Values lower than 0.5 were suppressed.

**FIGURE 1:**
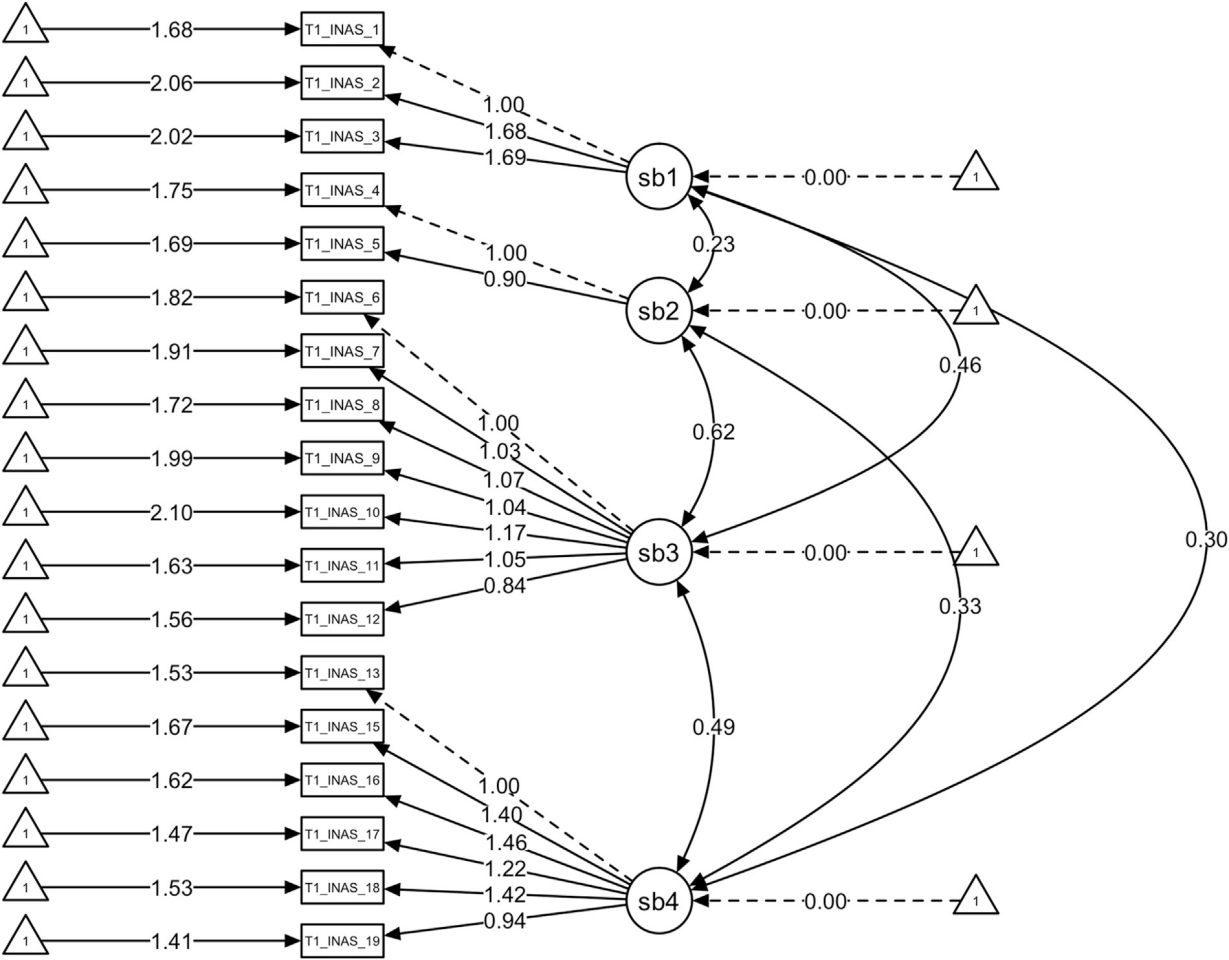
Confirmatory factor analysis using structural equations models for the portuguese version of INAS: models assessed and their respective fit indexes.

Confirmatory factor analysis, testing the four-factor model, was also performed using SEM techniques and the results are presented in Figure 1. The RMSEA is 0.132, showing a good fit for the model (0.12–0.15). The remaining metrics, although not reaching the ideal adjustment value (CFI > 0.9 and TLI > 0.9), are very close (CFI > 0.855 and TLI > 0.828) and support good to moderate fit for the model.

### Construct (Convergent and Discriminant) Validity

Taking into account the theoretical model on which the INAS is based, we defined a set of previously described hypotheses regarding the interrelations between the INAS and other instruments (see *Materials and Methods*), presented in Table 3. As expected, the results for adherence measured by MARS-9P had a negative correlation with all subscales of INAS. The newly emerged subscales of “resisting treatment” and “mistrust treatment” had strong positive correlations with BMQ scales of Harm, Overuse, and Concerns. A negative correlation between the Necessity subscale and the Resisting Treatment subscale was obtained. No correlations were found between the BMQ subscales and the Testing Treatment subscale. A significant positive correlation was obtained between the BMQ Overuse and Concerns subscales and the INAS Resisting Illness subscale. Finally, significant positive correlations between pain beliefs and all INAS subscales were obtained. Concerning the PBPI subscales, the results showed positive correlations between Mystery and all the INAS subscales. Constancy and Self-blame PBPI subscales revealed strong positive correlations with Resisting Treatment and Resisting Illness INAS Subscale, respectively. Finally, the all correlations were weak or moderate.

**TABLE 3:** Convergent and discriminant validity of INAS: Person correlation coefficients between subscales of the MARS-9P; subscales of BMQ; and PBPI global and subscales.

	INAS subscales			
Scales and subscales	Testing treatment	Mistrust treatment	Resisting treatment	Resisting illness
MARS-9P	−0.476*^b^*	−0.248*^b^*	−0.549*^b^*	−0.446^**^
BMQ	—	—	—	—
Harm	0.007	0.219*^a^*	0.241*^b^*	0.164
Overuse	0.077	0.335*^b^*	0.274*^b^*	0.203^*^
Necessity	0.059	−0.162	−0.179*^a^*	−0.037
Concerns	0.118	0.259*^b^*	0.284*^b^*	0.344^**^
PBPI	—	—	—	—
Mystery	0.183*^a^*	0.265*^b^*	0.250*^b^*	0.284^**^
Permanence	−0.004	−0.009	0.073	0.025
Constancy	0.097	0.157	0.256*^b^*	0.142
Self-blame	0.121	0.001	0.091	0.248^**^
PBPI_Global	0.177*^a^*	0.190*^a^*	0.283*^b^*	0.320^**^

Medication adherence rating scale-9 Portuguese (MARS-P9), Brief Medication Questionnaire (BMQ), and Portuguese Brief Pain Inventory (PBPI).

a Significant correlation at significance level *p* < 0.001.

b Significant correlation at significance level *p* < 0.05.

### Differences in Intentional Non-Adherence Scale Subscales Between Adherence Patterns During 1 Month

Considering the adherence behavior pattern at the two assessment moments, 39% of patients change their adherence behavior (from adherent to nonadherent or from nonadherent to adherent), 31% were adherent during one month, and 30% reveal a nonadherence behavior. At the baseline, there is a propensity to nonadherents testing (*p* = 0.001), mistrust (*p* = 0.002), resisting treatment (<0.001), and also resisting illness (*p* = 0.001). After one-month treatment, those who changed the adherence pattern tend to test the treatment more (0.02) and nonadherents continue to resist treatment.

## Discussion

This study aimed to present the first translation and cultural adaptation for the Portuguese language of the newly and, to the best of our knowledge, only instrument to explain different aspects of intentional nonadherence—the INAS, in patients suffering from CP. Accordingly, given the proportion of the patient population who do not adhere to treatments, it is a challenge to understand the complexity of its determinants. Moreover, the importance of unintentional factors, such as forgetting (Lehane and McCarthy, 2007; Gadkari and McHorney, 2012), is well recognized, and intentional factors related to patient’s beliefs and motivation variables have been documented in recent literature (Mukhtar et al., 2014; Sampaio et al., 2020). The protocol for translation, cultural adaptation, and validation was performed as outlined, developing a validated Portuguese version of INAS showing good psychometric properties. Internal consistency measured by Cronbach’s alpha coefficient was excellent (*α* = 0.902) for the total scale. Concerning the Resisting Illness subscale, the result for consistency was slightly below the original, *α* = 0.889 compared to *α* = 0.950, and equally excellent for Testing Treatment subscale α = 0.930) (Weinman et al., 2018). Two additional subscales emerged from the factor analysis of INAS (Weinman et al., 2018), which, although not in accordance with the original two-factor structure, was included after careful consideration and meetings with the experts and with the concordance of the authors of INAS. Thus, internal consistency was excellent for both of the newly emerged subscales: the Mistrust treatment subscale (*α* = 0.930) and the Resisting Treatment subscale (*α* = 0.917). These subscales must be interpreted within the patient’s view about their adherence behavior to CP treatment (Butow and Sharpe, 2013), which tends to be mainly due to intentional resistance and mistrust of medicines for CP treatment, as revealed in a recent study (Sampaio et al., 2020). Moreover, our results also emphasize that nonadherence to treatments is common in CP patients (MacPherson, 2002; Sampaio et al., 2020) and that a change of the adherence behavior is a reality in a short period of time, such as 1 month. This result may explain the poor retest reliability coefficients for all INAS subscales. The efficacy of pain medicines to persistent pain is limited (Broekmans et al., 2009), which may also compromise adherence behavior.

The present study used the same criteria to assess the validity of the Portuguese version of the scale as the original INAS validation (Weinman et al., 2018). Unsurprisingly, strong negative correlations were obtained between the two scales measuring adherence in CP patients (INAS and MARS-P9), similar to what was found in the United Kingdom Oncology and Hypertension samples (Weinman et al., 2018). Like in the original INAS study, no correlations were found between the BMQ Necessity subscale and the INAS subscales (Weinman et al., 2018). An exception was a moderate correlation between the newly emerged Resisting Treatment INAS subscale and the BMQ Necessity subscale, which may be related to a disbelief about the real effectiveness of the CP treatment, previously associated with analgesics usage (Lin et al., 2006; Sampaio et al., 2020). For the BMQ Concerns subscale, significant strong correlations were obtained for the original INAS Resisting Illness subscale and the two new subscales, emphasizing the worries that CP patients have about their medicines, which are reflected in the INAS items. Although the Testing Treatment INAS subscale did not correlate with other BMQ subscales, which may be due to the awareness of CP patients of the possible side effects of analgesic drugs (Sampaio et al., 2020). In the present study, the authors opted for including two BMQ dimensions (not used in the original validation study of INAS), which could inform about the validity of the newly subscales. Accordingly, both subscales have strong to moderate associations with the Harm and Overuse BMQ subscales, which may reflect an inherent belief about possible side effects of drugs and the perception that physicians over-prescribe medicines (World Health Organization, 2003). It has well recognized the role that beliefs and perceptions have in the physical and psychological adjustment and in the consequent treatment management in patients affected by CP (Williams et al., 1994; Stroud et al., 2000). Thus, it is important to use a Portuguese adapted and validated instrument to measure pain-related beliefs and perceptions (Azevedo et al., 2017). Significant correlations were obtained between pain beliefs and all INAS subscales. Interestingly, the Mystery PBPI subscale correlated significantly with all INAS subscales, which is consistent with the poor understanding of the problem of CP with clear consequences on intentional adherence behavior. Similar to the original validation study (Weinman et al., 2018), we ensured that INAS subscales were sufficiently distinct from other self-regulatory constructs of pain perception, such as Permanence, Constancy, and Self-blame. There were only two exceptions: first, the association between the perception of the constancy of CP and the resistance to treatment, which may emphasize that the problem of nonadherence also occurs in symptomatic conditions, like CP (Haynes et al., 1996) and, second, the link of the self-blame belief about CP with the resistance to illness, as if adherence to treatments remind patients of their own CP condition, which could prompt an emotional response that is avoided indirectly by nonadherence behaviors.

We believe that assessing different aspects of intentional nonadherence to pain medicines, using INAS as a self-report measure in clinical practice, is simple and can inform the patient’s own perspective, precluding some previously clinical judgment (Osterberg and Blaschke, 2005). The concepts captured by the Portuguese version of INAS could be used to inform targeted treatment approaches, as suggested by Weinman et al. (2018). Concomitantly, it will be a challenge to foster novel approaches to the adherence determinants revealed by the Portuguese version of INAS in CP patients.

This study must be interpreted taking into account its main limitations. Two additional factors emerged to explain different aspects of intentional nonadherence in CP, which need further evaluation; also, the educational level in our sample, that is, the real reflection of the educational level of the elderly population in Portugal. Following this, the questionnaires were filled in by the interviewer which could influence some of the responses. The final model shows a good adjustment using RMSE metric, but the remaining metrics, namely, CFI and TLI, did not reach the ideal adjustment value but are very close. This is a limitation which could be related to the small sample size. Models with small to modest sample sizes and maximum likelihood (ML) estimation of confirmatory factor analysis (CFA) models can show serious estimation problems, such as nonconvergence or parameter estimates, that are outside the admissible parameter space (Smid et al., 2020). Despite these limitations, the present study had strengths worth noting. First, it applied a rigorous methodological approach that followed the best internationally recommended standards for the adaptation and validation of health measurement instruments. Second, it is the first known evaluation of reliability and validity in a different clinical population in other country and cultural setting.

In conclusion, INAS has demonstrated to be adequate and to have some excellent psychometric characteristics. Although the newly emergent INAS subscales do not fit exactly with original factor structure, the Portuguese version of INAS could be a valuable and available instrument for Portuguese researchers and clinicians to assess the intentional nonadherence determinants during the management of chronic pain. There is an urgent need to characterize different “phenotypes” of nonadherence which could create a strong basis for future patient tailored interventions.

## Data Availability

The original contributions presented in the study are included in the article/Supplementary Material; further inquiries can be directed to the corresponding author.
